# Acupuncture for diabetic peripheral neuropathy: study protocol for a randomized, placebo-controlled trial

**DOI:** 10.1186/s13063-020-04811-3

**Published:** 2020-10-26

**Authors:** Haiping Deng, Yu Shu, Peiran Lv, Ling Zhao, Ke Cheng, Tingting Zhang, Yi Song, Hua Yang, Hong Tang, Jian Pei, Xueyong Shen

**Affiliations:** 1 School of Acupuncture-Moxibustion and Tuina, Shanghai University of Traditional Chinese Medicine, Pudong District, Shanghai, China; 2 Shanghai Research Center of Acupuncture and Meridian, Pudong District, Shanghai, China; 3 Long-Hua Hospital affiliated to Shanghai University of Traditional Chinese Medicine, Xuhui District, Shanghai, China

**Keywords:** Acupuncture, Diabetic peripheral neuropathy, Randomized controlled trial, Study protocol

## Abstract

**Background:**

Diabetic peripheral neuropathy (DPN) is the most common chronic complication of diabetes mellitus that has a considerable impact on quality of life, but there are few effective therapeutic strategies. The aim of this trial is to determine the efficacy and safety of manual acupuncture (MA) versus sham acupuncture (SA) for DPN.

**Methods/design:**

This is a study protocol for a randomized, placebo-controlled clinical trial. A total of 118 patients with DPN will be recruited and randomly assigned in a 1:1 ratio to either the MA group or SA group. All patients will receive 24 sessions over 12 weeks. Participants will complete the trial by visiting the research center at month 6 for a follow-up assessment. The primary outcome is peroneal motor nerve conduction velocity (peroneal MNCV) at week 12 compared with baseline. Secondary outcomes include peroneal motor nerve action potential amplitude (peroneal MNAP) and latent period (peroneal MNLP), sural sensory nerve conduction velocity (sural SNCV), action potential amplitude (sural SNAP) and latent period (sural SNLP), fasting plasma glucose (FPG), 2-h postprandial blood glucose (2hPG), glycated hemoglobin (HbAlc) at week 12 compared with baseline, Michigan Neuropathy Screening Instrument (MNSI) score and Diabetes Specific Quality of Life scale (DSQL) at week 12 and month 6 compared with baseline. Safety will be assessed during the whole trial. Masking effectiveness will be assessed by patients.

**Discussion:**

This trial may provide high-quality evidence for evaluating the efficacy and safety of MA treatment for DPN compared with SA treatment. Results of this study will be published in peer-reviewed journals.

**Trial registration:**

Chinese Clinical Trials Registry ChiCTR1800020444. First registered on 29 December 2018, retrospectively registered, http://www.chictr.org.cn/showproj.aspx?Proj=31063.

## Background

Diabetic peripheral neuropathy (DPN) is the most common cause of neuropathy worldwide, and its prevalence increases with the duration of diabetes [1]. Approximately 50% of patients with diabetes will develop peripheral neuropathy [1–3]. DPN is symmetric and predominantly sensory, starting distally and gradually spreading proximally in a glove-and-stocking distribution [1]. DPN is associated with the development of foot problems such as foot ulcers and is one of the leading causes of amputations. The high-frequency underreport and undertreatment of DPN can lead to an increased risk for morbidity and mortality and decreased quality of life [1, 2]. Once symptoms appear, there are few effective therapeutic strategies [4]. While neuropathic pain and paresthesia can be palliated by anti-convulsant drugs, tricyclic antidepressant drugs, or serotonin-noradrenalin re-uptake inhibitors [5], pharmacological management of decreased sensation is generally ineffective. Therefore, more and more patients turn to seek non-pharmacological treatments. Multiple complementary and alternative medicine therapies such as acupuncture and yoga have shown efficacy in the treatment of painful peripheral neuropathy [6]. A pilot RCT has demonstrated practicality and feasibility of acupuncture as an additional well-tolerated treatment without appreciable side effects for people with DPN [7]. Acupuncture may effectively ameliorate selected DPN symptoms, such as aching pain, burning pain, prickling sensation, numbness, and allodynia in American Indian patients [8]. Reviews concerning acupuncture for peripheral neuropathy (PN) have found that, despite the majority of studies reporting positive results, a reliable statement of effectiveness is not possible due to methodological limitations such as unsuitable control group and blinding [9–11]. Also, inconsistency of outcome measures and lack of use of global neuropathy-specific patient-reported outcome measures have confounded results. Overall, the quality of acupuncture intervention delivered in individual studies was low. Appropriately powered RCTs with suitable control groups, validated and proper outcome measures for DPN patients, and more accurate reporting of acupuncture interventions, including point selection rationales, point locations, and practitioner qualifications, are needed. Therefore, we have designed a randomized, sham-controlled, participant-blinded and assessor-blinded study with a 6-month observation period to explore its effectiveness and safety.

## Methods

### Study design

**Fig. 1:**
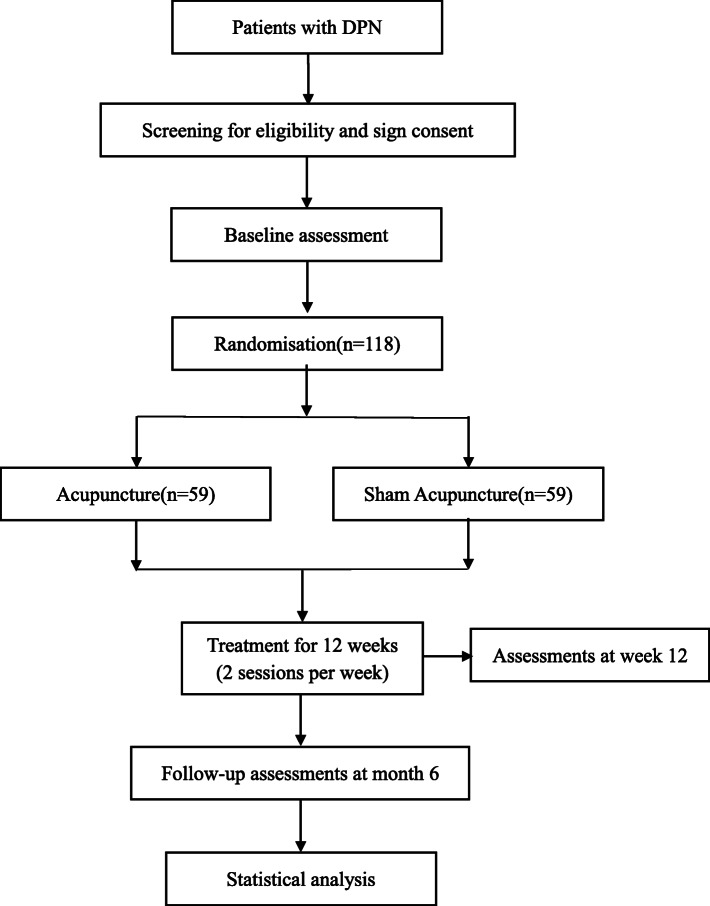
Flow diagram

This randomized, participant-blind, sham-controlled trial will be conducted at Long-Hua Hospital affiliated to Shanghai University of Traditional Chinese Medicine in China. The protocol for this trial is reported based on the Standard Protocol Items: Recommendations for Interventional Trials (SPIRIT) 2013 Checklist: defining standard protocol items for clinical trials (Additional file 1). The study has been approved by the Ethics Committee of Long-Hua Hospital (Ethical approval number: 2016LCSY028) and registered on the Chinese Clinical Trial Registry (Chictr) platform since 29 December 2018 (Registration number: ChiCTR1800020444). A flow diagram of the trial is shown in Fig. 1.

### Patient recruitment

Patients meet the American Diabetes Association (ADA) 2012 diagnostic criteria for DPN will be recruited from the endocrinology departments of Long-Hua Hospital affiliated to Shanghai University of Traditional Chinese Medicine in Shanghai, China. Recruitment posters will be posted in the hospital and uploaded to social media (WeChat) from October 2016 to December 2021. All patients will be required to provide written informed consent (Additional file 2) before randomization.

### Inclusion criteria

Participants who meet all the following requirements will be allowed for enrollment:

1. Aged 18–75 years (either sex)
2. Patients who meet diagnosis of DPN defined by ADA in 2005 [12]
  - DPN is defined as the presence of symptoms and/or signs of peripheral nerve dysfunction in people with diabetes, after the exclusion of other causes
  - NP is defined as altered sensitivity to pressure and (1) altered sensitivity to pain or (2) altered sensitivity to vibration or (3) achilles reflex [13]
3. Symmetric and predominantly sensory, starting from the lower limbs distally and gradually spreading proximally in a glove-and-stocking distribution
4. Ability to understand study procedures and willingness to comply with them for the entire period of study
5. Written informed consent

### Exclusion criteria

Participants meeting any of the following criteria will be excluded:

1. PN caused by conditions other than diabetes (e.g., alcohol abuse, chemotherapy, hereditary causes, chronic inflammatory, or idiopathic PN)
2. Psychiatric illnesses other than mild depression
3. Severe or unstable cardiovascular, liver, kidney, respiratory, or hematological disorders
4. Received acupuncture treatment in the last 3 months
5. Pregnant or lactating women
6. Research unit personnel directly related to the study and their immediate family members
7. Incapable of giving informed consent or following the study instructions due to language disturbances, serious cognitive deficits, or lack of time
8. Currently participating in other clinical trials

### Randomization and allocation concealment

All eligible patients will be randomly assigned to manual acupuncture (MA) group or sham acupuncture (SA) group in a 1:1 ratio. The blocked randomization sequence will be computer-generated (Excel) by an independent statistician, who will not be involved in implementation and statistical analysis of the trial. The allocation will be placed inside sequentially numbered sealed opaque envelopes which will be opened only after enrollment. The treatment allocation will be revealed to acupuncturists out of patients’ sight to ensure blinding. To reduce the risk of observer bias, the acupuncture practitioners will be discouraged from discussing treatments or previous results with patients. The random allocation sequence and sealed opaque envelopes will be kept separately by two specific researchers. The clinical research coordinator will be responsible for enrolling patients, obtaining informed consent, and requesting randomization.

### Masking

Due to the nature of acupuncture, masking of acupuncturists is quite difficult to achieve. Patients, outcome assessors, and statisticians who perform the statistical analyses will be blinded to group assignment. The treatments subjects received will be not revealed until the statistical analysis is completed.

### Interventions

Treatment will be performed by licensed acupuncturists who have at least 5 years of experience in acupuncture. All the acupuncturists will be trained how to locate acupoints, puncture, and manipulate needles before trials. A placebo device [14, 15] will be applied in both groups for better implementation of blindness. Both MA and SA treatments will consist of 24 sessions over 12 weeks (40 min per session, two sessions per week). Acupuncture will be discontinued if patients suffer from any serious adverse events (AEs).

### Manual acupuncture

**Fig. 2:**
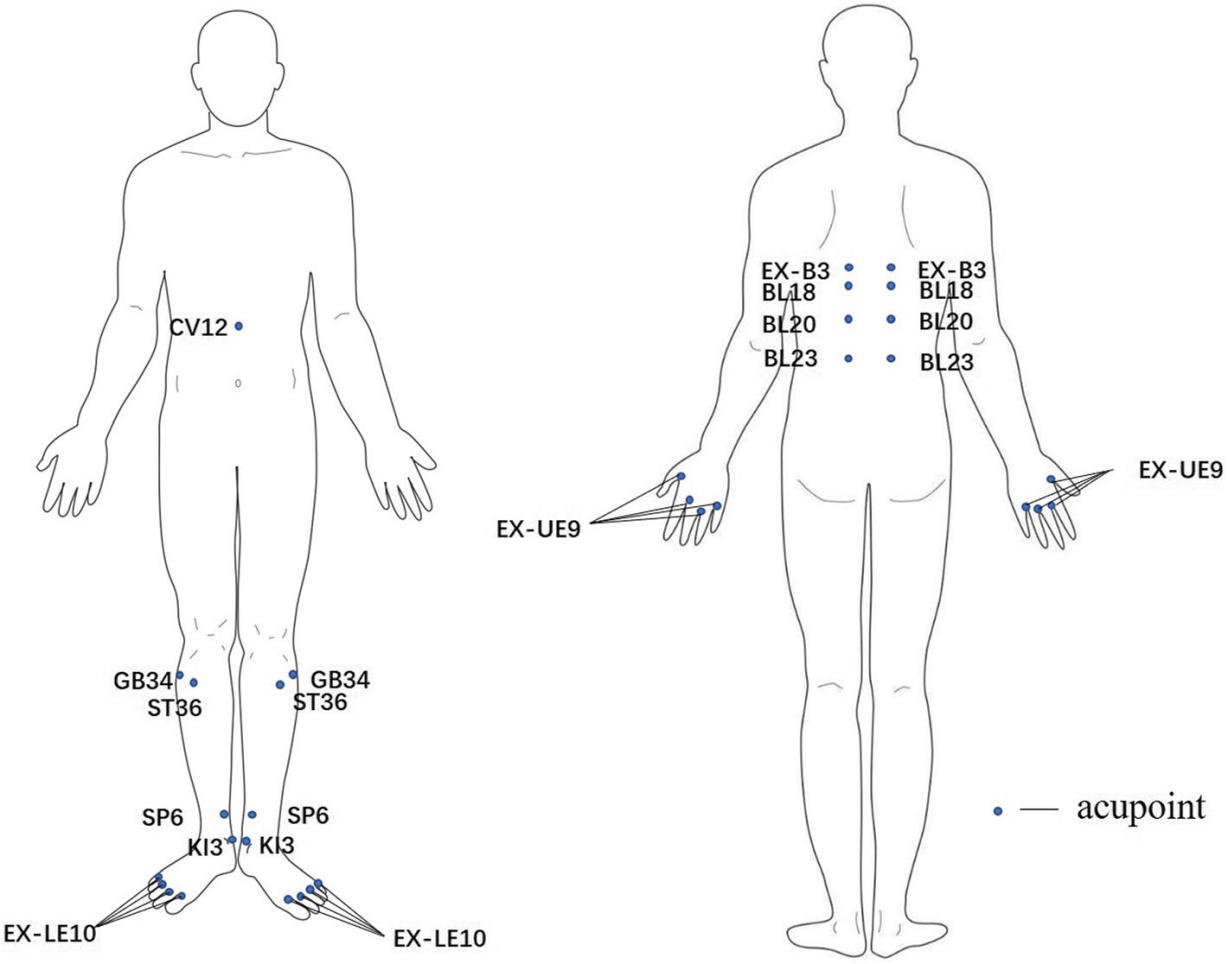
Locations of acupoints

Patients allocated to the MA group will receive treatment with needles being inserted at the prespecified acupuncture points through the placebo device. Obligatory and additional acupoints adopted in this protocol are developed from clinical experiences of acupuncture experts. The obligatory acupoints include Zhongwan (CV12), bilateral Weiwanxiashu (EX-B3), bilateral Ganshu (BL18), bilateral Pishu (BL20), bilateral Shenshu (BL23), bilateral Zusanli (ST36), bilateral Yanglingquan (GB34), bilateral Sanyinjiao (SP6), bilateral Taixi (KI3), and Bafeng (EX-LE10). Additional acupoints Baxie (EX-UE9) will be added when patients’ symptoms appear not only in the lower limbs but also in the upper limbs. All acupoints are localized according to the WHO Standard Acupuncture Locations and exhibited in Table 1 and Fig. 2. After skin disinfection, disposable, stainless steel acupuncture needles (0.25 × 25 mm or 0.25 × 40 mm, provided by Wuxi Jiajian Medical Instrument Co. Ltd., China) will be inserted into the skin of acupoint (approximately 10–20 mm depth), and then manipulations of twirling, lifting, and thrusting will be performed on all needles for at least 10 s to reach De qi (a compositional sensation including soreness, numbness, distention, and heaviness), which is believed to be an essential component for acupuncture efficacy. Firstly, CV12, ST36, GB34, SP6, KI3, EX-LE10, and EX-UE9 will be punctured when patients lie supine. Then, EX-B3, BL18, BL20, and BL23 will be punctured when patients lie prone. Needles will be retained in these acupoints for 20 min.

**Table 1:** Locations and manipulations of acupoints

	Acupoint	Location	Manipulation
Obligatory acupoints	Zhongwan (*CV12*)	On the anterior midline, 4 cun^a^ above the umbilicus	Puncture perpendicularly to a depth of 1.0–1.2 cun
	Weiwanxiashu (*EX-B3*)	at the same level as the inferior border of the spinous process of the 8th thoracic vertebra (T8), 1.5 cun lateral to the posterior median line	Puncture obliquely and medially to a depth of 0.5–0.8 cun
	Ganshu (*BL18*)	at the same level as the inferior border of the spinous process of the 9th thoracic vertebra (T9), 1.5 cun lateral to the posterior median line	Puncture obliquely and medially to a depth of 0.5–0.8 cun
	Pishu (*BL20*)	at the same level as the inferior border of the spinous process of the 11th thoracic vertebra (T11), 1.5 cun lateral to the posterior median line	Puncture obliquely and medially to a depth of 0.5–0.8 cun
	Shenshu (*BL23*)	at the same level as the inferior border of the spinous process of the 2nd lumbar vertebra (L2), 1.5 cun lateral to the posterior median line	Puncture perpendicularly to a depth of 0.8–1.2 cun
	Zusanli (*ST36*)	3 cun directly below Dubi (*ST35*), and one finger-breadth lateral to the anterior border of the tibia	Puncture perpendicularly to a depth of 1.0–1.2 cun
	Yanglingquan (*GB34*)	in the depression anterior and distal to the head of the fibula	Puncture perpendicularly to a depth of 1.0–1.2 cun
	Sanyinjiao (*SP6*)	3 cun superior to the prominence of the medial malleolus, posterior to the medial border of the tibia,	Puncture perpendicularly to a depth of 1.0–1.2 cun
	Taixi (*KI3*)	in the depression between the prominence of the medial malleolus and the calcaneal tendon	Puncture perpendicularly to a depth of 0.5–0.8 cun
	Bafeng (*EX-LE10*)	on the dorsum of the foot, between the first and fifth toes at the junction of the red and white skin posterior to the margin of the web; 4 points on each foot, 8 in total	Puncture obliquely to a depth of 0.5–0.8 cun
Additional acupoints for upper limbs syndrome	Baxie (*EX-UE9*)	at the dorsum of the hand, between the first and fifth fingers, proximal to the web margins between the five fingers at the junction of the red and white skin, 4 points on each hand,8 in total	Puncture obliquely upward to a depth of 0.5–0.8 cun

^a^1 cun (≈ 20 mm) is defined as the width of the interphalangeal joint of patient’s thumb

### Sham acupuncture

Patients in the SA group will receive sham acupuncture. The procedure and duration of treatment in the SA group will be identical in the MA group except the needles (0.25 × 25 mm, blunt tip, provided by Wuxi Jiajian Medical Instrument Co. Ltd., China) are blunt tip and there will be no skin penetration and needle manipulation for De qi.

Although it is difficult to set an eligible placebo control, using blunt-tipped needles that do not penetrate the patient’s skin is the most common approach for administering sham treatments among acupuncture trials, according to a literature review [16, 17]. Moreover, this study will exclude those who have received acupuncture treatment in the last 3 months and can distinguish SA from MA. The same control with no skin penetration at acupoints was adopted and succeeded in masking patients with DPN [7, 14, 15, 18]. Furthermore, all patients will be asked to guess which treatment they have received to test the patient-blinding effects at week 12.

### Concurrent treatments

Participants will receive routine health care as provided to all other patients with DPN. These treatments include glucose control, antihypertensive therapies, dyslipidemia control, analgesic, and neurotrophic treatments, if necessary. All information regarding the use of medications (including date of administration, types, and dosage) will be recorded. This will contribute to improving adherence.

### Follow-up

After 12-week treatments, all participants will enter an additional 3 months follow-up period. During this time, they will receive routine health care as provided to all other patients with DPN. However, acupuncture treatment will not be permitted during follow-up.

### Outcomes

#### Primary outcome

The primary outcome is peroneal motor nerve conduction velocity (peroneal MNCV) which will be assessed at baseline and at week 12 after randomization.

#### Key secondary outcomes

Secondary outcomes include peroneal motor nerve action potential amplitude (peroneal MNAP) and latent period (peroneal MNLP), sural sensory nerve conduction velocity (sural SNCV), action potential amplitude (sural SNAP), and latent period (sural SNLP). The outcome measurements will be assessed at baseline and at week 12 after randomization.

Additional secondary outcomes include glycated hemoglobin (HbA1c), fasting plasma glucose (FPG), 2-h postprandial blood glucose (2hPG), body mass index (BMI), and blood pressure (BP). The outcome measurements will be assessed at baseline and at week 12 after randomization.

### Michigan Neuropathy Screening Instrument (MNSI) score

The MNSI is a clinical and semi-quantitative evaluation of neuropathy that includes medical history and physical assessment. Medical history will be completed by patients with scores ranging between 0 and 13. Physical assessment will be completed by health professionals with five indicators and the aggregate score ranging between 0 and 10: foot appearance (0 and 1 for normal and abnormal, respectively), ulceration (0 and 1 for normal and abnormal, respectively), ankle reflex (0, 0.5, and 1 for normal, reenforced, and absent, respectively), vibration test (0, 0.5, and 1 for normal, weakened, and absent, respectively), and monofilament examination (0, 0.5, and 1 for normal, weakened, and absent, respectively) of feet on both sides. The outcome measurement will be assessed at baseline, at week 12 and at month 6 after randomization.

### Quality of life (QoL)

Disease-specific QoL will be assessed at baseline and at week 12, month 6 after randomization using Diabetes Specific Quality of Life scale (DSQL). The scale consists of four domains: interference (12 items), psychology (8 items), social relations (4 items), and treatment (3 items). Each item is measured with a 5-point Likert scale ranging from “not at all” to “extremely”. Higher scores indicate worse QoL.

### Patients’ global assessment of DPN

Patients’ global assessment of DPN will be evaluated on a 5-point Likert scale at week 12. Patients will be asked to respond to the following question: “Considering all the ways your DPN affects you, how are you doing today?” 1 = very poor; 2 = poor; 3 = fair; 4 = good; or 5 = very good.

### Blinding assessment

To test the patient-blinding effects, all patients will be asked to guess their group assignment allocation within 2 min after the last treatment session at week 12 as following: “Which group do you think you are in?” A. traditional acupuncture; B. modified acupuncture; or C. not sure.

### Credibility and expectancy

Credibility and expectancy of patients will be assessed by a Likert-scale Credibility/Expectancy Questionnaire (see Table 2) before the first treatment.

**Table 2:** Expectations for the efficacy of acupuncture are varied. If using following statements to describe your views on your disease/symptoms after the entire course of treatment, how much do you agree? For each statement, please choose the closest answer

	Strongly disagree	Disagree	Neutral	Agree	Strongly agree
a. My illness will get much better.	□_1_	□_2_	□_3_	□_4_	□_5_
b. I will face my disease better.	□_1_	□_2_	□_3_	□_4_	□_5_
c. Symptoms of my disease will disappear.	□_1_	□_2_	□_3_	□_4_	□_5_
d. I will be more vigorous than before.	□_1_	□_2_	□_3_	□_4_	□_5_

### Adverse events

AEs data will document the occurrence, duration, and severity of adverse reactions (symptoms and signs), and how the event was resolved (or not) during the treatment. Based on their potential association with the acupuncture needling procedure, AEs will be categorized by acupuncturists and related specialists as treatment-related or not within 24 h after occurrence. Common treatment-related AEs include local subcutaneous hematoma, itching at the sites of needle insertion, continuous post-needling pain, dizziness, and so on. All participants will receive routine blood test, liver function (alanine transaminase and aspartate transaminase), and kidney function tests (serum creatinine and blood urea nitrogen). These tests will be performed twice, after randomization and at the end of 12-week treatment period. Serious adverse events will be reported to Medical Ethics Committee and the participant will be treated with relevant conventional therapy or hospitalization if necessary (the participant’s allocated intervention will be revealed).

### Assessment of safety

Patients will be asked to assess safety of treatments after 12 weeks’ treatments, using a four-grade scale: safe, relatively safe, unsafe, and very unsafe.

### Sensation during the treatment

Patients will be asked about their sensation during each treatment period as following: “What sensation do you feel during the treatment? A. soreness; B. distention; C. pain; D. no feeling; E. else, please specify___.”

### Usage of medication

Medication Usage Log: Participants will be given a printed log to record their daily intake of prescribed medications. We will measure outcomes at week 12.

**Fig. 3:**
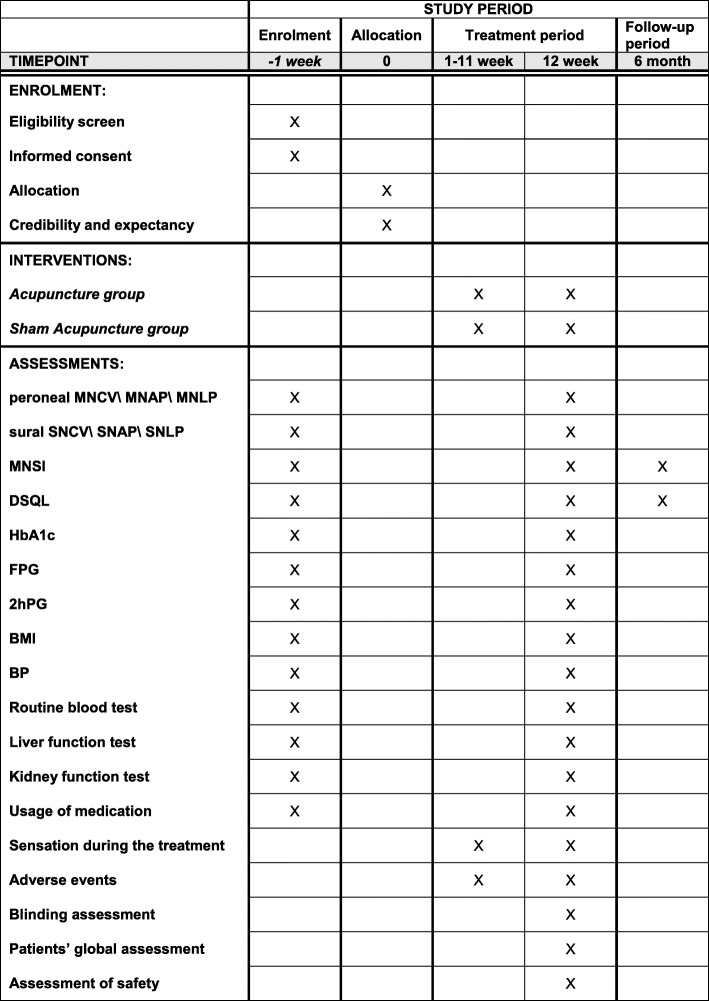
The schedule of enrolment, interventions, and assessments

The schedule of enrollment, interventions, and assessments are presented in Fig. 3.

### Data collection, management, and monitoring

All researchers including acupuncturists, outcome assessors, and statisticians will receive training regarding data management. Case Report Forms (CRFs) will be completed and double entered into the Electronic Data Capture (EDC) system by two independent investigators to ensure accuracy of data.

All research documents, including both paper files and electronic documents, will be preserved for at least 5 years after publication. If reviewers or readers have any questions regarding our published data, they can contact the corresponding author for access to original data or visit ResMan (http://www.medresman.org/uc/project/projectadd.aspx). Private information of patients including name, telephone number, and ID number will be anonymous to ensure participant confidentiality.

Safety of this study will be monitored by a team consists of independent clinical experts and statisticians with access to unblinded data from Data and Safety Monitoring Board (DSMB) of the clinical evaluation center of Long-Hua Hospital, affiliated to Shanghai University of TCM. DSMB is independent from the sponsor, the competing interests, and the investigational site and will review the performance and safety of the trial every 6 months.

The criteria for unblinding and discontinuing allocated interventions for any trial participant experiencing serious acupuncture-related AEs have been described previously. DSMB will reveal a participant’s allocated intervention and make the final decision on whether to terminate the trial.

### Statistical methods

#### Sample size

Based on results of a previous study, a sample size of 47 participants per group was estimated to provide 80% power to detect a between-group difference of 2.4 m/s of conduction velocity improvement of common peroneal nerve, with a pooled standard deviation of 4.15 m/s and an alpha level of 0.05. The assumption of a conservative 20% drop-out rate in any group increases the sample size to 59 participants per group or 118 participants in total.

#### Statistical analysis

Statistical analysis will be performed by an independent statistician who is not aware of group allocation. SPSS 21.0 (IBM SPSS Statistics, New York, USA) will be used for data analysis. Level of significance will be established at α < 0.05 with a two-sided test. Continuous data will be represented by mean ± standard deviation or median (range), and categorical data will be represented by percentage. All efficacy analyses will be performed using the intent-to-treat (ITT) approach. For the ITT analysis, population will consist of all patients who have been randomized. Missing data will be imputed using the last observation carried forward (LOCF) method. Continuous variables will be compared using Student’s *t* test or Wilcoxon rank-sum test as appropriate. Categorical variables will be compared using Fisher’s exact test or Wilcoxon rank-sum test as appropriate. Analysis of covariance (ANCOVA) or logistic regression will be used for analysis and adjustment of baseline characteristics that differ significantly between two groups.

#### Ethics and dissemination

The study protocol which follows principles of the Declaration of Helsinki has been approved by the Medical Ethics Committee of Long-Hua Hospital affiliated to Shanghai University of Traditional Chinese Medicine since 7 July 2016 (Approval Number: 2016LCSY028) and registered on the Chinese Clinical Trial Registry, ChiCTR1800020444. Results will be disseminated through peer-reviewed publications, a master’s thesis, or conference presentations. Data will be anonymized prior to publication to prevent identification of individual participants.

## Discussion

Diabetes mellitus (DM) is a common disease which is accompanied by highly significant social and economic burdens [19]. According to the International Diabetes Federation (IDF), DM affects 415 million people worldwide, with a prevalence of 8.8%, and these values are expected to rise still further to 642 million by the year 2040, with a prevalence of 10.4% [20]. DPN is one of the most frequent complications of DM and usually characterized by gradually increases in pain severity, impairments in tactile and proprioceptive sensation, vibration sense, and improper postural control [21]. DPN is commonly associated with high rates of mortality and morbidity [22]. Acupuncture has been widely used in clinical practice for the treatment of DPN in China. However, so far there has been no appropriately designed randomized controlled trial to provide clear evidence about the effectiveness of acupuncture treatment for DPN at home and abroad.

In this study, the inclusion criterion of age, from 18 to 75 years, was chosen to cover age range as wide as possible. We cooperated with the endocrinology department of Long-Hua Hospital affiliated to Shanghai University of Traditional Chinese Medicine to ensure the required number of subjects recruited. During the study period, patients will receive routine health care as provided to all other patients with DPN. Usually, we encourage them not to change their medications, but adjusting medications will be permitted if necessary. We believe this strategy can reflect real-world practice and satisfy ethical obligations better.

A standardized treatment protocol will be utilized to assure reproducibility of the study. In this trial, the treatment protocol was based on traditional acupuncture theory, previous studies [15], and the consensus of endocrinologists and acupuncturists from Long-Hua Hospital affiliated to Shanghai University of Traditional Chinese Medicine. The amount of acupuncture is intensive, which is similar to clinical practice in China. Needles will be stimulated manually for at least 10 s at each acupoint and retained in place for 20 min. There will be 2 treatment sessions per week within the course of 12-week treatment, giving a total of 24 sessions.

A suitable control group is critical for a well-designed clinical trial. On the basis of literature review, using a blunt-tipped needle that does not penetrate the patient’s skin is the most commonly used approach for administering sham treatments among acupuncture trials [16, 17]. Patients who have received acupuncture treatment in the last 3 months and can distinguish SA from MA will be excluded. The same control group design, no skin penetration at acupoints, was adopted and succeeded in masking patients with DPN [7, 14, 15, 18]. Furthermore, all patients will be asked to guess which treatment they have received to test patient-blinding effects at week 12.

The change of nerve conduction velocity is chosen as the primary outcome because it is the highest sensitive and objective method to detect neuropathy. Multiple systematic reviews [11] recommended nerve conduction velocity as a main outcome, and it had been widely used as an outcome measure in DPN studies [23]. At the same time, assessment of the severity of DPN mainly depends on patient-reported outcomes. The MNSI is the most DPN-specific patient-reported measure. In this trial, we use the DSQL to evaluate quality of life in patients with DPN before and after acupuncture treatment. The DSQL captures the impact of detailed aspects of modern diabetes management (e.g., carbohydrate counting and flexible insulin dose adjustment) that is extensively used in evaluating quality of life in DM. The DSQL offers a valuable tool for assessing the impact of treatment approaches on QoL in adults with DM [20].

The limitation of our trial is acupuncturists are not blinded for the nature of intervention. We hope results of this trial will provide more reliable evidence and clarify the value of acupuncture as a treatment for DPN.

To summarize, this trial meets the methodological demand of adequate randomization and allocation concealment, blinding of patients, outcome assessors, and statisticians. Findings of this study will provide high-quality evidences for evaluating the efficacy and safety of acupuncture treatment for DPN.

## Trial status

This trial is currently recruiting patients. This is version 2.0 of the protocol, dated June 6, 2016. Recruitment began on 10 October 2016 and the anticipated end date is 31 December 2021.

## Supplementary information

**Additional file 1.** Completed Standard Protocol Items: Recommendation for Interventional Trials (SPIRIT) 2013 Checklist: items addressed in this clinical trial protocol.

**Additional file 2.** Informed consent.

## Data Availability

All de-identified data collected during the trial will be available for anyone who wishes to access 6 months after publication in accordance with FAIR principles.

## Abbreviations

ADA: The American Diabetes Association
AEs: Adverse events
BMI: Body mass index
BP: Blood pressure
CRF: Case report form
DM: Diabetes mellitus
DPN: Diabetic peripheral neuropathy
DSMB: Data and Safety Monitoring Board
DSQL: Diabetes Specific Quality of Life scale
FPG: Fasting plasma glucose
HbAlc: Glycated hemoglobin
2hPG: 2-h postprandial blood glucose
IDF: International Diabetes Federation
ITT: Intent-to-treat
MA: Manual acupuncture
MNAP: Motor nerve action potential amplitude
MNCV: Motor nerve conduction velocity
MNLP: Motor nerve action potential latent period
MNSI: Michigan Neuropathy Screening Instrument
PN: Peripheral neuropathy
QoL: Quality of life
SA: Sham acupuncture
SNAP: Sensory nerve action potential amplitude
SNCV: Sensory nerve conduction velocity
SNLP: Sensory nerve action potential latent period
