# Supplementary material for: Acupuncture for diabetic peripheral neuropathy: study protocol for a randomized, placebo-controlled trial
Source: Trials. 2020 Oct 26;21:879. doi: 10.1186/s13063-020-04811-3 (PMC7586683; doi:10.1186/s13063-020-04811-3)
Supplement: Supplementary file 2 — Additional file 2. Informed consent. [file 13063_2020_4811_MOESM2_ESM.pdf]

陆氏针灸治疗糖尿病周围神经病变的随机盲法对照临床试验

受试者知情同意书—告知页

尊敬的患者：

我们将邀请您参加 “上海市中医药事业发展三年行动计划海派中医流派传承工程”的临床研究，评估陆氏针灸治疗糖尿病周围神经病变的的临床疗效。

在您决定是否参加这项研究之前，请仔细阅读以下内容，它可以帮助您了解该项研究以及为什么要进行这项研究、研究的程序和期限，参加研究后可能给您带来的益处、风险和不适。

以下是本项研究的介绍：

一、研究背景

糖尿病周围神经病变（DPN）是糖尿病最常见的慢性并发症之一，无论 1 型或 2 型糖尿病，神经病变的发生率均很高，据调查有 10%-50%的糖尿病患者发生 DPN，且随糖尿病病程的延长，其发病率呈明显上升趋势。对于该病的治疗，现代医学除控制血糖外，多应用 B 族维生素、血管扩张剂、镇痛药物等，其疗效不甚满意。针灸在治疗该病方面积累了丰富的经验，相关报道也很多，但由于诊断标准及疗效标准不一，样本含量过少，从而使临床疗效难以肯定。因此，对针灸治疗糖尿病周围神经病变开展高质量的随机对照研究非常必要。本项目欲开展随机盲法对照试验，以客观评价针灸治疗糖尿病周围神经病变的疗效。

本试验已通过上海中医药大学附属龙华医院医学伦理委员会审议，认为是遵从我国法律法规和赫尔辛基宣言，符合医学伦理道德的。

本试验将在上海中医药大学附属龙华医院针灸科进行，届时将有至少118名患者参加。

二、哪些人不宜参加本项研究

1）患有躁狂症、抑郁症等精神病；

2）患有严重的或不稳定的心血管、肝脏、肾脏、呼吸系统或血液系统疾患、有症状的外周血管疾病；

3）研究者认为在研究过程中会影响参加研究或可能导致住院的其他躯体疾病或心理疾病；

4）曾使用过明确会引起神经病变的药物（如长春新碱），；

5）曾患有可能会引起神经病变的疾病，如恶性贫血和甲状腺机能减退；

6）患有颈腰椎病变、脑梗塞或格林--巴利综合征；

7）妊娠、哺乳期妇女；

8) 无法完成各种量表填写；

9）不愿意被随机分组；

10）同时参与其他研究。

三、如果参加研究需要做什么

在入选本项研究前，您将接受以下检查以确定是否可以参加研究：①医生将询问、记录病史、进行体格检查；②密西根神经病变筛选量表（MNSI）以确诊，糖尿病患者生存质量特异性量表（DSQL），并测神经传导速度、空腹血糖、餐后 2h 血糖、糖化血红蛋白、血常规、肝肾功能以及患者对治疗反应的总体评价。

如果您符合入选条件，您将纳入研究。研究开始将根据计算机提供的随机数字，决定您接受传统针刺或安慰针刺治疗。参加这项研究的患者分别有 50%的可能性被分入这两个不同的治疗组。您和您的医生都无法事先知道和选择任何一种治疗方法。为了避免心理等其他因素的影响，您在整个疗程中将不被告知属于哪一种治疗组，请理解。

我们将根据您的病变部位选取穴位，进行针刺治疗，每次治疗 40 分钟， 2 次/周，共治疗 12 周，共计 24 次。治疗过程中如果您因故不能按时治疗，请务必在当周内补做治疗。

您需要在初次治疗前及治疗结束后填写量表和问卷，共 2 次，请务必如实填写并独立完成。若您无法到医院，我们将电话访问您或邮寄问卷给您，请您及时填写并回寄给研究组。

您在治疗前及治疗后所抽取的静脉血样，将用于检测空腹血糖、餐后 2h 血糖、糖化血红蛋白、血常规及肝肾功能。

需要您配合的其它事项：

请您按医生和您约定的访视时间来医院就诊，这对于本项研究非常重要，因为医生将判断您所接受的治疗是否真正起到作用。

四、研究中可能的受益

您和社会将可能从本项研究中受益。此种受益包括您的病情有可能获得改善，以及本项研究可能帮助开发出一种新治疗方法，以用于患有相似病情的其他病人。

五、参加研究可能的不良反应、风险和不便

针刺不良反应和针刺意外较常见的有血肿、剧烈疼痛、后遗不适感和金属过敏，较严重的为晕针、感染、气胸、神经损伤和内脏损伤。

如果在研究中您出现任何不适，或病情发生新的变化，或任何意外情况，不管是否与针刺有关，均应及时通知您的医生，他/她将对此作出判断和医疗处理。

医生将尽全力预防由于本研究可能出现的不良反应。如果在临床试验中出现不良反应，课题组将对试验相关的、需要诊治的不良反应提供治疗的费用及相应的经济补偿，这一点已经在我国《药品临床试验质量管理规范》中做出了规定。

您在研究期间需要按时到医院接受治疗，这些都可能给您造成麻烦或带来不方便。试验结束后，申办方将提供一定的交通补助。

六、有关费用

本课题组将支付您参加本项研究期间所做的与研究有关的检查费用，随访时的挂号费，并免费提供针刺治疗，分次给予交通补贴，共计 500 元/人。

如果发生与试验相关的损害，本课题组将支付您的医疗费用，并提供适当的营养费、就诊的交通费、误工的工资和奖金的补偿费。如果您同时合并其他疾病所需的治疗和检查，将不在免费的范围之内。

七、个人信息的保密

您的医疗记录（包括 CRF 及理化检查报告）将按规定保存。研究者、申办者代表、伦理委员会将被允许查阅您的医疗记录。任何有关本项研究结果的公开报告将不会披露您的个人身份。我们将在允许的范围

内，尽一切努力保护您个人医疗资料的隐私。

除本研究以外，有可能在今后的其他研究中会再次利用您的医疗记录。您现在也可以声明拒绝除本研究外的其它研究利用您的医疗记录。

八、您可以获得更多的信息

您可以在任何时间提出有关本项研究的任何问题。医生将给您留下医院和自己的电话号码以便能回答您的问题。

如果在研究过程中有任何重要的新信息，可能影响您继续参加研究的意愿时，医生将会及时通知您。

九、可以自愿选择参加研究和中途退出研究

是否参加本项研究完全取决于您的自愿。您可以拒绝参加此项研究，或在研究过程中的任何时间退出研究，这都不会影响您和医生的关系，并且不会遭到歧视和抱负，您的医疗待遇与权益不受影响。

医生出于对您的最大利益考虑，可能会随时中止您参加本项研究。

如果不参加本项研究，或中途退出，还有很多其它替代的治疗药物。您不必为了治疗您的疾病而必须选择参加本项研究。

如果您因为任何原因从研究中退出，您可能被询问有关使用试验药物情况。如果医生认为需要，您也可能被要求进行实验室检查和体格检查。

如果您选择参加本项研究，我们希望您能够坚持完成全部研究过程。

十、现在该做什么

是否参加本项研究由您自己决定。您可以和您家人或者亲友讨论后再做出决定。

在您做出参加研究的决定前，请尽可能向医生询问有关问题，直至您对本项研究完全理解。

感谢您阅读以上材料。如果您决定参加本项研究，请告诉医生，医生会为您安排一切有关研究的事务。

请您保留这份资料。

受试者知情同意书—签字页

患者声明

1、我已阅读了上述有关本研究的介绍，而且有机会就此研究与医生讨论并提出问题。我提出的所有问题都得到了满意的答复。

2、我知道参加本研究的受益和风险。我知道参加研究是自愿的，我确认已有充足的时间对此进行考虑，而且明白：

（1）我可以随时向医生咨询更多的信息；

（2）我可以随时退出本研究，而不会受到歧视和报复，医疗待遇与权益不会受到影响；

（3）我同样清楚，如果我中途退出研究，特别是由于治疗的原因使我退出研究时，我应将病情变化告诉医生，完成相应的体检和理化检查，这将对我和整个研究十分有利；

（4）如果因病情变化需要采取任何其他的药物治疗，我会在事先征求医生的意见，或在事后如实告诉医生；

（5）我同意监督管理部门，伦理委员会或申办者代表查阅我的研究资料；

（6）我同意（ ）或拒绝（ ）除本研究以外的其它研究利用我的医疗记录。

最后，我确认已了解本试验的详细情况，包括其权利以及可能的受益和风险。我决定同意参加本项研究，并保证尽量遵从医嘱。同时，我已经得到一份双方签名的知情同意书副本。

受试者签名：____________________________

联系电话：______________________________ 日　　期：______年____月____日

医生声明

我确认已向患者解释了本研究的详细情况，包括其权利及可能的获益和风险，并给其一份签署过的知情同意书副本。

医生签名：_____________________________

联系电话：_____________________________ 日　　期：______年____月____日

如您对该研究的伦理有任何疑问可以电话上海中医药大学附属龙华医院医学伦理委员会联系方式：021-64385700 转 1318
